# NIR Sensing Technologies for the Detection of Fraud in Nuts and Nut Products: A Review

**DOI:** 10.3390/foods13111612

**Published:** 2024-05-22

**Authors:** Miguel Vega-Castellote, María-Teresa Sánchez, Irina Torres-Rodríguez, José-Antonio Entrenas, Dolores Pérez-Marín

**Affiliations:** 1 Department of Bromatology and Food Technology, University of Cordoba, Rabanales Campus, 14071 Córdoba, Spain; g32vecam@uco.es; 2 Department of Animal Production, University of Cordoba, Rabanales Campus, 14071 Córdoba, Spain; g72toroi@uco.es (I.T.-R.); p82enlej@uco.es (J.-A.E.)

**Keywords:** food authentication, detection of adulterants, near-infrared spectroscopy, hyperspectral imaging, targeted and non-targeted approaches

## Abstract

Food fraud is a major threat to the integrity of the nut supply chain. Strategies using a wide range of analytical techniques have been developed over the past few years to detect fraud and to assure the quality, safety, and authenticity of nut products. However, most of these techniques present the limitations of being slow and destructive and entailing a high cost per analysis. Nevertheless, near-infrared (NIR) spectroscopy and NIR imaging techniques represent a suitable non-destructive alternative to prevent fraud in the nut industry with the advantages of a high throughput and low cost per analysis. This review collects and includes all major findings of all of the published studies focused on the application of NIR spectroscopy and NIR imaging technologies to detect fraud in the nut supply chain from 2018 onwards. The results suggest that NIR spectroscopy and NIR imaging are suitable technologies to detect the main types of fraud in nuts.

## 1. Introduction

Food fraud is a long-standing issue that has increased in occurrence in recent years given the sophisticated nature of the food supply chain and the growth of dishonest practices of reducing production costs or deceiving costumers regarding product quality to obtain a higher market price. In Regulation (EU) 2017/625 [1], the European Union (EU) established the general rules in official controls to identify risks associated with the use of products, processes, or materials that could have a negative influence on the integrity of foodstuffs and to account for any information regarding the composition, properties, origin, or production method that could mislead consumers. This regulation arose as a consequence of the billions of euros that the fraudulent practices cost the EU every year and the effects that these practices can have on consumers’ health, the quality of the commercialized foodstuffs, and on public confidence in the agri-food chain [2]. Although efforts have been made to counter food fraud [see, for example, the FoodIntegrity European Project (ID: 613688), with participants from 18 European countries, which aims to ensure the quality, safety, and authenticity of the European food chain], there is unfortunately still a long way to go to reduce and prevent fraud in the agri-food chain.

The Knowledge Centre for Food Fraud and Quality (KC-FFQ) of the European Commission provides expertise for policy making, creating a network of food fraud experts [3] and publishing the ‘Monthly Food Fraud Summary Reports’. In these reports, several cases of food fraud related to nuts have been published in the past few years, such as the reported lack of documentation of 98 tons of almonds sold as organic, valued at EUR 753,000 in Italy in 2020, or the incorrect labelling of pistachio nuts in France and Spain, valued at EUR 6 million, in 2021 [4]. According to the Combined Nomenclature (CN) code provided in Regulation (EU) 2017/1925, the fruits most commonly included in the ‘nuts’ category are almonds, Brazil nuts, cashew nuts, chestnuts, hazelnuts, macadamia nuts, peanuts, pecan nuts, pine nuts, pistachio nuts, and walnuts [5]. It is key to note that although peanuts are, in botanical terms, strictly legumes, they are commonly included in the nuts group, as indicated by Buthelezi et al. [6]. Nuts are high-added-value products with a total production over 1 million tons in the EU in 2022 [7] and are a valuable source of energy, healthy fats, proteins, fiber, and vitamins. They are commonly sold as whole nuts or as ingredients in other formats, e.g., ground, to be processed by the manufacturing industry. Fraudulent practices in nuts are a major issue that can involve economic fraud and even pose a threat to human health [8,9], and for this reason, there is an urgent need to make reliable and rapid techniques available to assist in fraud detection throughout the food supply chain [10]. 

A wide range of analytical techniques have been proposed to assure the authenticity of foodstuffs [11,12]. In nuts, the most common ones are those based on chromatographic techniques [13,14,15], polymerase chain reaction (PCR) methods [16,17], spectroscopic technologies [18,19,20,21,22], and mass spectrometry fingerprinting alternatives [23,24] among others. The chromatographic techniques and PCR methods require the preparation of the sample prior to analysis, which involves destroying the original structure of the product. In addition, these methods entail a high cost per analysis, are time-consuming, require trained staff, and are not suitable for assessing large volumes of product, which, in many cases, is a key factor in identifying fraud in foodstuffs. However, spectroscopy-based applications offer the possibility of assuring the authenticity of nuts in a fast and non-destructive way while analyzing large amounts of product. Among these, the most popular technique for fraud detection in nuts is near-infrared (NIR) spectroscopy [25]. This technique is based on the absorbance that takes place when a molecule bond vibrates at the same specific frequency as the incident NIR light irradiating the product. A wide range of applications can be found using NIR spectroscopy in nuts [6,26], such as the successful quantitative estimation of the lipid oxidation in in-shell and shelled hazelnuts, carried out by Pannico et al. [27], or the qualitative application for the identification of commercial sweet almond batches adulterated with bitter almond kernels, carried out by Torres-Rodríguez et al. [28], which showed an excellent capability for discrimination. Additionally, the detection of adulterated products with other non-compliant ingredients, e.g., different nuts and fragments of nutshells or non-edible elements, could benefit from the combination of conventional spectroscopy and imaging techniques, providing not only the spectral information but also the spatial properties of the commodity analyzed [29]. NIR imaging technology offers the possibility to obtain both spectral and spatial features of an object, integrating the power of spectroscopy and digital imaging, which can be of great utility in specific applications and complex issues. This technology is known as hyperspectral imaging (HSI) if the image is composed of hundreds of contiguous wavebands and as multispectral imaging (MSI) if only a few spectral bands (usually between three and ten bands) are included in the spectral dimension [30,31]. 

In the scientific literature, we found only one review (Teixeira & Sousa [25], submitted for publication in May 2018) that focuses on NIR applications for origin authentication and the detection of adulteration in nuts. Thus, the aim of this work is to provide a review on the application of NIR-based techniques for the detection of fraud in nuts and nut products over the last 7 years (2018–2024).

## 2. Bibliographic Search

**Figure 1:**
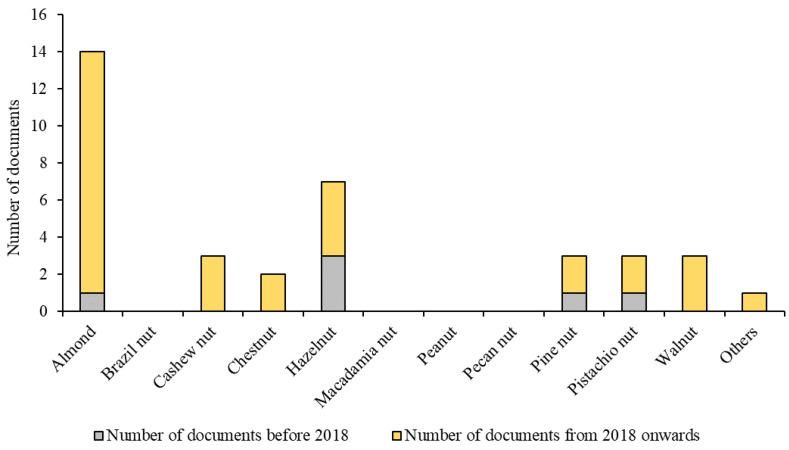
Numbers of studies related to food fraud detection in nuts before 2018 and from 2018 onwards.

The literature review was carried out using three scientific databases, Scopus, Web of Science, and Google Scholar. The keywords used (in different combinations) included the range of nuts considered—‘almonds’, ‘Brazil nuts’, ‘cashew nuts’, ‘chestnuts’, ‘hazelnuts’, ‘macadamia nuts’, ‘peanuts’, ‘pecan nuts’, ‘pine nuts’, ‘pistachio nuts’, ‘walnuts’, and the term ‘nut’—together with the techniques reviewed (in this case, ‘NIR spectroscopy’ and ‘hyperspectral imaging’) and words that made any reference to fraud, such as ‘adulteration’, ‘authentication’, ‘fraud’, and ‘counterfeit’. Once the list of potential research papers to be included in the review was made, we used the following criteria to accept or reject a study: (1) Assurance of the quality of the documents—only peer-reviewed papers published in journals included in the Journal Citation Reports (JCR) index were considered. The peer-review criterion was also used for books and chapters of books. (2) Nuts were presented in intact, ground, powder, or paste formats, excluding other presentations such as nut oils. (3) Only NIR-based techniques were used, excluding other spectroscopic techniques such as mid-infrared or Raman spectroscopy. (4) Any reference to the authentication of nuts or issues of fraud needed to be reported in the document in question. For example, a document would be accepted if the objective was the classification of pistachio cultivars to detect fraud in their commercialization; however, a paper would not be considered in this review if the objective was the classification of pistachio cultivars per se, with no mention or consideration of food fraud. (5) The earliest date for a document to be included in this review was set at 2018. The number of documents published in the past 7 years regarding fraud detection in nuts using NIR spectroscopy and HSI was significantly greater than the number of documents available in the years before 2018 (Figure 1). Consequently, this review is of great importance to summarize the research information available on this topic from the past seven years.

## 3. The Nature of Fraud in Nuts and Types of NIR Applications Reviewed

Nowadays, several approaches to food fraud can be taken due to the great complexity of the food supply chain and the latest trends in which foodstuffs are produced, processed, distributed, and sold [32]. Momtaz et al. [33] indicated the main mechanisms of food adulteration, including the adulteration of food properties by adding artificial coloring agents or preservatives and the substitution of a specific food ingredient by adding another ingredient of inferior quality and value. Moreover, other types of fraud related to authentication issues can be found in the scientific literature, such as the mislabeling of foodstuffs, which takes advantage of the high economic value of products of a superior quality category or those that have been produced in specific regions and associated with a Protected Geographical Indication (PGI), Protected Designation of Origin (PDO), or similar quality standards [34]. In nuts, different type of fraud can be found depending on the format of the commercialized product. One of the most common types identified in the papers we reviewed was the adulteration of ground, powder, or paste nut products by using other foodstuffs of a lower value, including different nuts or fruit, such as the adulteration of almond powder with apricot or peanut powder [35,36]. This can mainly lead to food safety issues (allergies) and consumer dissatisfaction, given the lower quality of the product they purchase and the financial deceit involved. In addition, although it is not so common, given the ease of committing fraud in the previously mentioned nut formats, several cases can be found of authenticity issues with intact nuts, including the year of stock, type of production (for example, organic farming) or origin, or their adulteration, such as the commercialization of non-compliant intact nuts. In this context, of the studies reviewed in this work (Table 1 and Table 2), three main targets were identified related to food fraud detection in different formats of nuts using NIR-based techniques: the identification of their origin, the authentication of the cultivar, and the detection of adulterants in the commercialized product. 

The origin of nuts is a key issue affecting the characteristics of their products since factors such as the climatologic conditions of the area or the soil properties and the management of the orchards have a significant influence on the chemical composition of the nuts and, thus, on their quality and final market price. Biancolillo et al. [37] and Sammarco et al. [38] developed methods to authenticate different PDOs and PGIs of Italian hazelnuts harvested in specific locations such as the Roma and Viterbo (Lazio, Italy) areas. The authentication of the origins of different nuts was also evaluated in the studies carried out by Firmani et al. [20] for almonds, Amendola et al. [39] for walnuts, and Nardecchia et al. [40] for chestnuts. Furthermore, nut cultivars are also a key factor in the market value of the product since its shape, taste, and/or aroma can differ from one cultivar to another. Hence, different studies have been carried out in nuts such as almonds, chestnuts, and pine nuts [41,42,43,44]. 

Finally, regarding the detection of adulterants in the commercialized product, a wide range of adulterants can be found in the studies reviewed, such as was suggested by the presence of green peas in pistachio nut products, given the lower price of the former [45,46]; the presence of bitter almonds in batches of sweet almonds, which can affect consumer safety [47,48]; the adulteration of cashew nuts with other allergenic nuts [49,50]; and the detection of foreign bodies in Chinese hickory nuts, such as shell fragments that can be harmful for human consumption [51]. To address these issues, different chemometric approaches were tested in the reviewed documents by means of the development of quantitative and qualitative models to detect fraud in nuts. The former aim at the estimation of the quantity of the undesired component in the adulterated product while the latter apply methods designed for the classification of the product according to its membership to a certain class. 

**Table 1:** Applications for fraud detection in nuts using NIR point spectroscopy techniques. N: number of samples; Ref.: reference.

Type of Nut	Aim of the Study	Sample Presentation and Analysis	Technique	Acquisition Mode	Spectral Range (nm)	Chemometric Technique	N	Performance	Ref.
Almonds	Cultivar authentication	Intact shelled. Two spectra per kernel.	NIR spectroscopy	Reflectance	1000–1700	Partial least squares–discriminant analysis (PLS-DA) and quadratic discriminant analysis (QDA)	120	Test set: 94% of almonds correctly classified	[41]
Almonds	Origin authentication (Avola almonds from Syracuse area, southeast of Sicily, Italy)	Intact. Two spectra per sample.	Fourier-Transformed (FT) NIR spectroscopy	Reflectance	1000–2500	PLS-DA and soft independent modeling of class analogies (SIMCA)	227	Test set: 95% of almonds correctly classified	[20]
Almonds	Origin authentication	Intact and ground. Up to six spectra per sample.	FT NIR spectroscopy	Reflectance	866–2532	Support vector machine (SVM)	64	Nested cross-validation: 62.6% of intact almonds and 79.1% of ground almonds correctly classified	[52]
Almonds	Detection of adulterants	Powder. One spectrum per sample.	FT NIR spectroscopy	Reflectance	1000–2500	Data-driven SIMCA (DDSIMCA) and one-class PLS (OCPLS)	260	Test set: 91–100% of adulterated samples correctly classified	[35]
Almonds	Cultivar authentication	Ground, five spectra per sample.	FT NIR spectroscopy	Reflectance	866–2532	SVM	250	Nested cross-validation: 80.3% of almonds correctly classified	[42]
Almonds	Detection of adulterants	Intact shelled almonds.	NIR spectroscopy	Reflectance	908–1676	PLS-DA	216	Cross-validation: 90% samples correctly classified	[28]
Almonds	Detection of adulterants	Intact shelled almonds.	NIR spectroscopy	Reflectance	950–1650	PLS-DA	216	Test set: 98.6% samples correctly classified	[28]
Almonds	Detection of adulterants	Intact in-shell and shelled. Batch spectra in dynamic mode. Four spectra per sample.	NIR spectroscopy	Reflectance	950–1650	PLS-DA	145	Test set: 95 and 100% of in-shell and shelled samples, respectively, correctly classified	[47]
Almonds	Detection of adulterants	Intact in-shell and shelled. Batch spectra in dynamic mode. Four spectra per sample.	NIR spectroscopy	Reflectance	908–1676	PLS-DA	145	Test set: 100% of in-shell and shelled samples correctly classified	[47]
Almonds	Detection of adulterants	Intact in-shell and shelled. Batch spectra in dynamic mode. Four spectra per sample.	NIR spectroscopy	Reflectance	950–1650	Modified PLS (MPLS) regression, LOCAL algorithm	145	Test set MPLS: R^2^ = 0.53 for in-shell samples; R^2^ = 0.96 for shelled samples. Test set LOCAL: R^2^ = 0.98 for shelled samples	[47]
Almonds	Detection of adulterants	Intact in-shell and shelled. Batch spectra in dynamic mode. Four spectra per sample.	NIR spectroscopy	Reflectance	908–1676	MPLS regression, LOCAL algorithm	145	Test set MPLS: R^2^ = 0.53 for in-shell samples; R^2^ = 0.96 for shelled samples. Test set LOCAL: R^2^ = 0.95 for shelled samples	[47]
Almonds	Cultivar authentication	Intact shelled. Batch spectra in dynamic mode. Four spectra per sample.	NIR spectroscopy	Reflectance	950–1650	Principal component analysis (PCA), Shewhart control chart, GH distance	140	Test set: 52–90% of almond batches correctly classified	[53]
Almonds	Cultivar authentication	Intact shelled. Batch spectra in dynamic mode. Two spectra per sample.	FT NIR spectroscopy	Reflectance	834–2502	PCA, Shewhart control chart, GH distance	140	Test set: 57–100% of almond batches correctly classified	[53]
Almonds	Origin authentication	Powder. Five spectra per sample.	FT NIR spectroscopy	Reflectance	866–2532	Linear discriminant analysis (LDA)	72	Nested cross-validation: 92.5–95.0% of almonds correctly classified	[54]
Almonds	Detection of adulterants	Powder. Three spectra per sample.	NIR spectroscopy	Reflectance	900–1700	DDSIMCA, SIMCA and OCPLS	182	Test set: 98.5% of almond samples correctly classified	[55]
Almonds	Detection of adulterants	Powder. Three spectra per sample.	NIR spectroscopy	Reflectance	950–1650	DDSIMCA, SIMCA and OCPLS	182	Test set: 98.5% of almond samples correctly classified	[55]
Almonds	Detection of adulterants	Powder. Three spectra per sample.	FT NIR spectroscopy	Reflectance	1350–2500	DDSIMCA, SIMCA and OCPLS	182	Test set: 96.4% of almond samples correctly classified	[55]
Almonds	Detection of adulterants	Powder. Three spectra per sample.	FT NIR spectroscopy	Reflectance	900–2500	DDSIMCA, SIMCA and OCPLS	182	Test set: 100% of almond samples correctly classified	[55]
Almonds	Detection of adulterants	Ground.	NIR spectroscopy	Reflectance	908–1676	SIMCA, decision tree (DT), logistic regression (LR), naive Bayes (NB), SVM, k-Nearest Neighbor (KNN), Gaussian Process (GP)	120	Test set: 100% of almond samples correctly classified	[36]
Almonds	Detection of adulterants	Ground.	FT NIR spectroscopy	Reflectance	1000–2500	SIMCA, DT, LR, NB, SVM, KNN, GP	120	Test set: 100% of almond samples correctly classified	[36]
Almonds	Detection of adulterants	Ground.	NIR spectroscopy	Reflectance	908–1676	PLS regression	120	Test set: R^2^ = 0.96 for the determination of adulterant concentration	[36]
Almonds	Detection of adulterants	Ground.	FT NIR spectroscopy	Reflectance	1000–2500	PLS regression	120	Test set: R^2^ = 0.96 for the determination of adulterant concentration	[36]
Cashew nuts	Detection of adulterants	Ground. One spectrum per sample.	NIR spectroscopy	Reflectance	908–1676	PLS-DA and SIMCA	280	Test set: 55.4–100% reliability rate	[49]
Cashew nuts	Detection of adulterants	Ground. One spectrum per sample.	NIR spectroscopy	Reflectance	908–1676	SIMCA	280	Test set: 95.7–98.7% of adulterated samples correctly classified	[50]
Cashew nuts	Detection of adulterants	Ground. One spectrum per sample.	NIR spectroscopy	Reflectance	908–1676	SIMCA	280	Test set: 95.7–100% of adulterated samples correctly classified	[56]
Chestnuts	Origin authentication (‘Vallerano’ chestnuts, Italy)	Intact in-shell. Four spectra on the pericarp and two on the hilum per individual fruit.	FT NIR spectroscopy	Reflectance	1000–2500	PLS-DA and SIMCA	441	Test set: 95% of in-shell and 97% of shelled walnuts correctly classified	[40]
Chestnuts	Cultivar authentication	Intact shelled. One spectrum per kernel.	FT NIR spectroscopy	Reflectance	1000–2500	PLS-DA	96	Cross-validation: 98% correctly classified	[43]
Hazelnuts	Origin authentication (Italian PDO ‘‘Nocciola Romana’’)	Intact in-shell. Two spectra per intact nut.	FT NIR spectroscopy	-	1000–2500	PLS-DA and SIMCA	376	Test set: 94% of hazelnuts correctly classified	[37]
Hazelnuts	Origin authentication (different regions of Turkey)	Intact in-shell and shelled. Two spectra per kernel.	FT NIR spectroscopy	Reflectance	1000–2500	PLS-DA and LR	280	Test set: 100% of in-shell and shelled hazelnuts correctly classified	[57]
Hazelnuts	Origin authentication (from five different countries)	Ground. Five spectra per sample.	FT NIR spectroscopy	Reflectance	866–2532	Discriminant classifiers using random subspaces for constructing DT	233	Nested cross-validation: 90.6% of hazelnuts correctly classified	[58]
Hazelnuts	Origin authentication (Italy: PGI from Piedmont and PDO from Campania)	Ground and paste. Fourteen spectra per sample.	NIR spectroscopy	Reflectance	400–2500	PLS-DA	216	Test set: 100% of intact hazelnuts correctly classified and 72% of paste hazelnuts correctly classified	[38]
Pine nuts	Cultivar authentication	Intact shelled. Two spectra per kernel.	NIR spectroscopy	Reflectance	1100–2300	PLS-DA and Interval-PLS-DA (iPLS-DA)	900	Test set: 97% of pine nuts correctly classified	[44]
Pine nuts	Cultivar authentication	Intact in-shell. One spectrum per kernel.	FT NIR spectroscopy	Reflectance	781–2632	DT, random forest (RF), MLP, SVM, NB	210	Test set: 99% of pine nuts correctly classified	[59]
Pistachio	Detection of adulterants	Ground. Three spectra per sample.	FT NIR spectroscopy	Reflectance	1250–2500	SIMCA	60	Test set: 100% of pistachio adulterated samples correctly classified	[46]
Pistachio	Detection of adulterants	Powder. Ten spectra per sample.	NIR spectroscopy	-	908–1695	PCA and PLS regression for adulterant quantification	143	PCA scores plot reported a clear grouping of classes. Test set PLS: R^2^ > 0.99	[45]
Walnuts	Origin authentication	Powder. One spectrum per sample.	FT NIR spectroscopy	Reflectance	833–2500	PLS-DA	555	Test set: 98.8–100% of walnuts correctly classified	[60]
Walnuts	Origin authentication (Sorrento area, south of Italy)	Intact in-shell and shelled. Two spectra per individual kernel.	NIR spectroscopy	-	-	PLS-DA	237	Test set: 95% of in-shell and 97% of shelled walnuts correctly classified	[39]
Walnuts	Origin authentication	Ground. Three spectra per kernel.	FT NIR spectroscopy	Reflectance	866–2532	LDA	212	Nested cross-validation: 77% of walnuts correctly classified	[61]

**Table 2:** Applications for fraud detection in nuts using hyperspectral imaging techniques. N, number of samples; Ref., Reference.

Type of Nut	Aim of the Study	Sample Presentation and Analysis	Technique	Acquisition Mode	Spectral Range (nm)	Chemometric Technique	N	Performance	Ref.
Almonds	Detection of adulterants	Intact shelled almond kernels	NIR HSI system	Reflectance	900–1700	PLS-DA	448	Test set: 97.1% of samples correctly classified	[62]
Almonds	Detection of adulterants	Intact shelled almond kernels	NIR HSI system	Reflectance	946–1648	PLS-DA	158	Test set: 75% of samples correctly classified	[48]
Chinese hickory nuts	Detection of adulterants	Intact shelled Chinese hickory nut kernels	Visible (VIS)–NIR HSI system	Reflectance	400–1000	PCA-KNN, and SVM	213	Test set: 99% of samples correctly classified	[51]

## 4. NIR-Based Instrumentation and Analysis Mode for the Detection of Fraud in Different Formats of Nuts

The wide range of applications for the detection of fraud in nuts using NIR-based technologies have been made possible due to the great development in terms of instrumentation over the last few years, enabling this technology to be used throughout the different steps of the food supply chain to meet the needs of producers, industry, retailers, and consumers [63,64,65]. In this work, the research studies reviewed were classified into two groups, according to the type of instrumentation used: conventional NIR spectroscopy instruments or multispectral/hyperspectral camera systems. 

### 4.1. NIR Spectroscopy

The versatility of NIR spectroscopy enables us to analyze nuts in the different formats available in the market such as intact, ground, powder, or paste, which can be considered as a major advantage for fraud detection in these products. This technology can be applied in various modes, namely reflectance, interactance, and transmission, using three main types of instruments, namely laboratory devices, instruments located in the sorting and grading industrial lines, and portable devices [66,67]. It permits the analysis of food products in the different steps of the supply chain, i.e., in the field (in-situ), in the laboratory (off-line), on the production line (at-line, on-line), etc.

The most common instruments used among the studies reviewed (Table 1) were those designed for at-line or off-line applications in different formats of nuts, such as the FT-NIR TANGO spectrometer (Bruker Optics, Bremen, Germany) [58,61] or the FT-NIR Nicolet 6700 spectrophotometer (Thermo Fisher Scientific, Madison, WI, USA) [20,37], which both work in reflectance mode. Although these FT-NIR laboratory devices offer high-spectral-resolution NIR measurements, their main disadvantage is that they can be only incorporated at-line and in the laboratory under controlled conditions and that the amount of product which can be inspected using them is limited compared to that using other types of instruments. In addition to the laboratory systems, other research papers used devices suitable for the on-line NIR analysis of nuts. One of the main advantages of this type of instrument is the possibility to analyze—directly from the processing line—greater amounts of product compared to the laboratory devices and, thus, to collect a greater variability, which is of major importance when dealing with food fraud detection. In this context, Vega-Castellote et al. [53] used the Matrix-F spectrophotometer (Bruker Optik GmbH, Ettlingen, Germany), which is an FT-NIR instrument interfaced to a fiber-optic NIR illumination and a detection head working in reflectance mode in the 834–2502 nm spectral range. The spectral range in which most of the benchtop and on-line instruments used in the studies we reviewed work (approx. 1000–2500 nm) provides key information for the detection of fraud in nuts. For example, the 1000–1100 nm band, at the beginning of the second overtone region, can be useful to discriminate hazelnuts of different geographical origins since that band can be associated with the presence of polyphenols in aromatic compounds [38] while the 1200 nm wavelength can be related to absorbances of the second overtone of C–H bonds of lipids [68]. Furthermore, the 1408–1650 nm region can be related to the N-H stretching (first overtone) of proteins, which can be useful for determining the origins of almonds [52]. In addition, the 1724 nm wavelength, linked to C-H stretching (first overtone), can provide information about the lipid contents of walnuts, which can be of great interest to identify the geographical origin of their products [61]. 

In addition, apart from these applications using laboratory and on-line NIR instruments, various research papers can be found that aimed to detect food fraud using portable NIR instruments. These devices belong to a new generation of NIR handheld instruments which permit the in-situ analysis of products at different steps throughout the food supply chain. Despite the reduced sizes of these instruments, the technological developments enable one to ensure the satisfactory performance of these devices [69]. The portable instruments used in the papers we reviewed work in reflectance mode in the spectral range of approx. 900–1700 nm so that key information for fraud detection in nuts can be extracted, as mentioned above. It is important to highlight that compared to on-line instruments, the area from which the NIR information is collected when using portable devices is smaller, given the limited window size of these devices. Nevertheless, different strategies to deal with this issue have been reported in the studies we reviewed, such as analyzing the nuts in dynamic mode (moving the portable sensor along the surface of the product) to collect the maximum amount of variability as possible, and to obtain more representative information from a sample [28], or taking more than one spectrum per sample, which, in addition, would enable one to guarantee the spectral repeatability of the data acquired. For example, Lösel et al. [54] acquired five spectra per powdered almond sample, and Arndt et al. [52] took six spectra per each intact almond sample. It is worth noting that even when the purpose is to obtain a representative measurement of a single kernel, it is of paramount importance to collect more than one spectrum per sample since, as reported by Nardecchia et al. [40], there is a difference in the spectral signatures of the measurements taken from different parts of a chestnut (pericarp versus hilum), which highlights the wide variability within a single kernel. In addition, these authors reported that taking the spectral information from the pericarp or the hilum could have an influence on the model’s performance when classifying PDO and non-PDO Italian ‘Vallerano’ chestnuts. 

### 4.2. NIR Imaging Systems

The studies available for fraud detection in nuts with NIR imaging devices all used hyperspectral systems (Table 2). These systems are generally composed of charged-coupled device (CCD) cameras, which include sensors with small photodiodes (pixels) made of materials sensitive to light such as silicon (Si) or indium gallium arsenide (InGaAs) [70], along with point, line, or area-scan spectrographs. The line-scan methods are well suited for taking information from moving samples by acquiring a slit of spatial and spectral information for each point in the linear field of view using conveyor belts or electric displacement platforms, which is of particular interest for analyzing products in food industry processing lines. These methods can be considered as extensions of the point-scan approach and are both known as spatial-scan methods [70]. For the line-scan systems, the speed of the displacement platforms is dependent on the experiment settings and may need to be adapted to industrial operation conditions when developing an application for routine analysis. Qin et al. [70] stated that HSI applications are the first step to determining the optimal wavebands with the aim of reducing the total volume of data to be used later as an MSI solution for a particular application. Faqeerzada et al. [62] used a commercial Pika NIR-640 (Resonon, Bozeman, MT) line-scan push-broom hyperspectral camera to acquire images in the 900–1700 nm range in reflectance mode in order to detect apricot kernels in almonds—the former is a cheaper product with similar color, texture, or odor compared to almonds—and reported excellent classification accuracy (~97%). Feng et al. [51] worked with an HSI system to identify shell fragments in Chinese hickory nuts. The authors reported that the color similarities between kernels and shell fragments can make it difficult to differentiate small but harmful endogenous foreign bodies in these nuts. To discriminate between these types of products, a line-scan HSI system consisting of a CCD camera working in reflectance mode in the VIS–NIR range (400–1000 nm) was used, which enabled the researchers to separate kernels from shell fragments with a high degree of accuracy (99%). Furthermore, Torres-Rodríguez et al. [48] also used a line-scan HSI system to detect bitter almonds in batches of sweet almonds by preparing mixtures in which the proportion of bitter kernels was increased from 5 to 20% in 5% steps. The system also featured a CCD camera working in the NIR range between 946 and 1648 nm, acquiring data in reflectance mode. Based on the results obtained in their study, the authors concluded that it would be possible to classify almonds by bitterness although further studies would be needed to be able to identify each individual bitter almond present in a mixture.

## 5. Strategies for the Treatment of the NIR Data and Chemometric Approaches

The complexity of the food matrices and the large number of data obtained when working with NIR-based techniques require using multivariate statistical tools for data interpretation. One of the first steps prior to the development of NIR models is to pretreat the spectral data in order to remove unwanted signals [71]. In addition, different chemometric approaches can be found to develop quantitative or qualitative applications to detect fraud in nuts. These approaches, in turn, can be targeted or not targeted, as explained below. 

### 5.1. Spectral Preprocessing of Nuts

In nuts, it is important to study the influence that the physical characteristics of the sample, like the shape of the nut or particle size (i.e., kernels analyzed in intact versus ground format), can have on the captured spectrum, given the absorbance displacement and the slope shift related to the scatter effect of the light when it interacts with a sample. In addition to the correction of the scatter effects—using, for example, standard normal variate (SNV) or multiplicative scatter correction (MSC) specifically suggested to eliminate drifts (non-linear baseline deviations) caused by scatter in reflectance measurements—other preprocessing methods should also be tested, such as the filtering and/or the scaling/centering signal pretreatments, since the latter, for example, often enables one to obtain a more simple and interpretable regression model [72]. Corona et al. [43] tested different pretreatments on the spectra of intact chestnuts such as SNV, MSC, and Savitzky–Golay (SG) derivatives to remove the unwanted spectral variance that could be associated with the variability in the refractive index and the morphology or density of the sample. These authors reported that the best modeling results were obtained using SNV in combination with derivatives. This pretreatment combination of scatter correction followed by a filtering method was the one most frequently used among the studies reviewed and could be seen in the research carried out by Moscetti et al. [44], Torres et al. [28], Vega-Castellote et al. [53], Shakiba et al. [58], and Netto et al. [55] among others. Likewise, Arndt et al. [61], who tested more than eight different pretreatments applied in different orders to classify walnuts analyzed in ground format based on their geographical origin, also concluded that the most suitable option to remove unwanted variations from the spectral data was the application of a scatter correction method—followed, in this case, by a centering pretreatment. It should be mentioned that while these studies selected the optimal pretreatment combination based on the models’ performance, none of them showed significance tests comparing the models’ performance when the different pretreatments were applied and, therefore, the fact that most of them used a scatter correction method along with other spectral pretreatments should be viewed with caution. Finally, other types of pretreatments can be found among the papers reviewed, such as the use of binning as a preprocessing technique to average the intensity of a different number of adjacent, strongly correlated wavenumbers to decrease the computational time of the spectral data [54]. 

### 5.2. Chemometric and Machine Learning Approaches for Fraud Detection in Nuts

Two types of chemometric approaches can be differentiated to deal with food fraud using NIR techniques. When the aim is the quantification of a specific compound/property of the matrix analyzed or the discrimination of previously defined classes, targeted chemometric methods are used. However, when the objective is to assess product deviations compared with a library of previously recorded compliant samples according to a standard (without focusing on any specific compound, but rather focusing on the whole fingerprint of the product in question), non-targeted methods are applied [73]. 

The targeted approach is the most traditional method in the NIR community for quality and safety determination in food products. Quantitative and qualitative applications are commonly carried out using regression and classification techniques such as the PLS regression or PLS-DA, respectively. However, non-targeted approaches are a recent, promising trend in NIR applications and have great potential to assure the integrity of food products. Many examples of non-targeted approaches can be found, such as the use of PCA or the OCPLS method [74,75].

#### 5.2.1. Targeted Approaches

The most frequently used approach to detect fraud in nuts is the application of the targeted linear PLS-DA technique (Table 1 and Table 2), for example, to detect adulterants in almonds [28] or cashew nuts [49], to authenticate the origin of almonds [20] or hazelnuts [37,38], and to authenticate the cultivars of almonds [41] or pine nuts [44]. One important aspect to take into consideration when using the PLS-DA technique, as mentioned by Vega-Castellote et al. [47], who assessed the presence of bitter almond kernels in commercial batches of sweet almonds, is the number of samples included per class since unbalanced models can bias the PLS-DA prediction boundary towards the smaller class. After the application of PLS-DA, different authors have proposed ways of identifying which variables have the greatest influence on the model using different methods such as Biancolillo et al. [37], who used the variable importance in projection (VIP) score values and found that the most important spectral regions for the origin authentication of hazelnuts were at approximately 1700–1785 nm, related to the presence of lipids, and 2000–2170 nm, associated with combination bands of the peptide bonds. The VIP values were also studied by Torres-Rodríguez et al. [48], who used a PLS-DA model to detect the presence of bitter almonds in batches of sweet almonds using an HSI system. The results obtained with the full spectral range (179 bands) and only the ones selected using the VIP values (25 bands) reported a similar accuracy, which is of special interest in HSI applications to reduce the amount of available data. 

In addition to PLS-DA, other chemometric targeted approaches have been used to detect fraud in nuts. Arndt et al. [61] used LDA to determine the geographical origin of walnuts. Prior to the application of this technique, these authors reduced the dimensions of the spectral data by applying PCA. Likewise, Cortés et al. [41] performed QDA to determine the cultivars of almonds in which, prior to the application of this algorithm, PCA was performed and the QDA was applied to the principal component scores obtained. When applying these types of algorithms (LDA or QDA), the dimension of the NIR data usually needs to be reduced—by using PCA or PLS, for example—since the number of variables must be lower than the number of samples [76]. 

Other approaches based on machine learning techniques have also been used such as the application of discriminant classifiers using random subspaces to construct DTs for the geographical determination of hazelnuts [58], in which the authors carried out a data fusion strategy using NIR and nuclear magnetic resonance (NMR) spectroscopies. Arndt et al. [52] and Arndt et al. [42] developed multiclass models to determine the geographical origin of almonds using SVM, which is a method that can be used to train linear and non-linear classifiers, for classification [77]. Huang et al. [59] also used machine learning methods, such as the RF, NB, or the multilayer perceptron (MLP), to identify the cultivars of pine nuts. Menevseoglu et al. [36] successfully identified ground almonds that had been adulterated with apricot kernels by applying different machine learning algorithms. The results obtained in these studies showed that the machine learning approaches, along with the NIR techniques tested, were effective means of geographical determination and cultivar identification in different nuts.

In addition to these classification techniques, quantitative approaches have also been tested in studies aiming at the detection of adulterants in nuts. Genis et al. [45] applied the PLS regression method to detect the presence of green peas and/or spinach powder in ground samples of pistachio, reporting a high degree of prediction accuracy. Vega-Castellote et al. [47] also used the MPLS regression technique and the LOCAL algorithm to identify adulterants in intact almonds by quantifying the amount of a toxic compound in the samples (Table 1). 

#### 5.2.2. Non-Targeted Approaches

There is a current and important discussion in the food authentication literature regarding the ‘discriminant’ and ‘class-modeling’ approaches [78]. Non-targeted techniques, which are focused on a single class and can be related to the class-modeling approach, can also be found among the studies reviewed. As can be seen from Table 1, the most frequently used is the SIMCA method. This class-modeling approach, based on PCA, models each of the classes individually, with new unknown samples accepted or rejected by each class model [79]. Firmani et al. [20] tested this method for the authentication of the origin of almonds, indicating the importance of presenting the results of the analysis in terms of sensitivity and specificity—samples correctly identified in their class and correctly rejected samples, respectively—given the fact that when the SIMCA method is used, one sample can be associated with more than one class. Biancolillo et al. [37] also applied the SIMCA method for to authenticate the origin of hazelnuts. In their study, they focused on a specific class (an Italian PDO hazelnut), with all the rest of the samples not belonging to that PDO assigned to a second class. This type of approach is known as asymmetric classification. In addition, other studies using the SIMCA method can be found for authenticating the origin of chestnuts [40], as well as for detecting adulteration in pistachios [46], cashew nuts [49], or almond flour [55] among others. In all cases, satisfactory results have been obtained using the SIMCA method. Nevertheless, it is important to note that in many studies in which PLS-DA and the SIMCA method were tested, the authors highlighted that PLS-DA outperformed the SIMCA method [40,49], given the fact that the SIMCA method models the intra-class variance, which is usually low, as was in the case of the study conducted by Miaw et al. [49], while PLS-DA maximizes the between-group variance [80]. An adaptation of the SIMCA method, the DDSIMCA method, was tested by Faqeerzada et al. [35] and Netto et al. [55] to detect adulterants in almonds in powder format. Both studies also applied the OCPLS algorithm, which is another non-targeted class-modeling method and which models only the target class and sets threshold boundaries during the development of the model. Although, in these studies, high accuracy (~90–100%) was obtained for the detection of adulterants in almonds in powder format, in both cases, it was reported that the DDSIMCA models performed better for classification when compared to OCPLS.

Another example of a non-targeted method was used by Vega-Castellote et al. [53], who developed a system based on PCA, Mahalanobis distances, and the construction of Shewhart control charts to detect non-compliant batches of sweet almonds, i.e., batches of sweet almonds adulterated with bitter kernels. A standard or control population of sweet almonds was characterized by means of PCA, and new adulterated samples (with 5, 10, 15, and 20% of bitter kernels) were projected into that PCA space. The distance (global Mahalanobis distance—GH) of each adulterated sample to the center of the control population was plotted on the Shewhart control charts and GH limits were set in order to detect the batches of adulterated almonds. The results obtained suggested that this approach, which uses solely the NIR spectral information of the product, would enable one to successfully perform conformity tests on batches of sweet almonds processed in the industry. 

## 6. Conclusions and Future Prospects

The adulteration of the physicochemical characteristics of nut products through the addition or substitution of any of their components not only has an economic impact –given the high value of this commodity—but can also have an impact on consumer health. In nuts, the main types of fraud are related to the identification of the origin, authentication of the cultivar, and the detection of adulterants in the commercialized product. The papers included in this review show that NIR technology is a suitable analytical alternative for the rapid detection of fraud in different nuts. The at-line or off-line applications were the most common type and were developed using laboratory devices that can be used in limited scenarios. However, only a few applications were available in the literature that use the new generation of portable and on-line NIR sensors to detect fraud in nuts. The feasibility of portable devices to perform NIR measurements in different steps of the food supply chain and the possibility of on-line sensors analyzing large amounts of produce, along with the need for fast, non-destructive analytical alternatives for routine operations to detect fraud in the food supply chain, mean there are promising perspectives for the application of these types of sensors in the upcoming years. In addition, HSI technology has become a powerful tool that combines characteristics of machine vision and point spectroscopy and is of great interest in the identification of fraud in intact nuts, where spatial information plays a significant role. Future prospects for this technology seek to improve the implementation of HSI systems by reducing the workload of imaging acquisition and processing so that real-time routine operations can be used for fast, non-destructive fraud detection and thus meet the needs of the nut industry. Although considerable advances have been achieved in the past few years in the field of HSI systems in terms of hardware and software, the speed of these systems is still the greatest handicap for their adoption in inspection processes in the food industry.

In the reviewed papers, different chemometric approaches were used to assess the occurrence of fraud in nuts, and these can be divided into targeted and non-targeted methods. Several papers included in this review used non-targeted methods, given that fraudsters often develop sophisticated alternatives to avoid detection through targeted methods. Thus, non-targeted methods are a promising tool for the screening of fraud in nuts, reducing the volume of product analyzed using confirmatory techniques. However, among the key challenges still to be addressed in the following years in order to implement these methods for fraud detection in nuts in routine operations in the industry are the development of legislative guidelines to apply these methods in routine analysis for official control and the validation of the models using independent sample tests on the industrial sorting lines.

## Data Availability

No new data were created or analyzed in this study. Data sharing is not applicable to this article.

## Nomenclature

CCD	charged-coupled device
CE	conditional entropy
CN	combined nomenclature
DDSIMCA	data-driven soft independent modeling of class analogies
DT	decision tree
EU	European Union
FT	Fourier-transform
GH	global Mahalanobis distance
GP	Gaussian process
HSI	hyperspectral imaging
InGaAs	indium gallium arsenide
iPLS	interval partial least squares discriminant analysis
JCR	journal citation reports
KC-FFQ	Knowledge Centre for Food Fraud and Quality
KNN	k-nearest neighbor
LDA	linear discriminant analysis
LR	logistic regression
MLP	multilayer perceptron
MPLS	modified partial least squares
MSC	multiplicative scatter correction
MSI	multispectral imaging
NB	naive bayes
NIR	near-infrared
NMR	nuclear magnetic resonance
OCPLS	one-class partial least squares
OJEU	official journal of the European Union
PCA	principal components analysis
PCR	polymerase chain reaction
PDO	protected designation of origin
PGI	protected geographical indication
PLS	partial least squares
PLS-DA	partial least squares–discriminant analysis
QDA	quadratic discriminant analysis
RF	random forest
SG	Savitzky–Golay
Si	Silicon
SIMCA	soft independent modeling of class analogies
SNV	standard normal variate
SVM	support vector machine
VIP	variable importance in projection
VIS	visible

## References

[1] Official Journal of the European Union (OJEU) (2017). Regulation (EU) 2017/625 of the European Parliament and of the Council of 15 March 2017 on official controls and other official activities performed to ensure the application of food and feed law, rules on animal health and welfare, plant health and plant protection products, amending Regulations (EC) No 999/2001, (EC) No 396/2005, (EC) No 1069/2009, (EC) No 1107/2009, (EU) No 1151/2012, (EU) No 652/2014, (EU) 2016/429 and (EU) 2016/2031 of the European Parliament and of the Council, Council Regulations (EC) No 1/2005 and (EC) No 1099/2009 and Council Directives 98/58/EC, 1999/74/EC, 2007/43/EC, 2008/119/EC and 2008/120/EC, and repealing Regulations (EC) No 854/2004 and (EC) No 882/2004 of the European Parliament and of the Council, Council Directives 89/608/EEC, 89/662/EEC, 90/425/EEC, 91/496/EEC, 96/23/EC, 96/93/EC and 97/78/EC and Council Decision 92/438/EEC (Official Controls Regulation) Text with EEA relevance. OJ L 95/1, 7.4.2017. https://eur-lex.europa.eu/legal-content/EN/TXT/PDF/?uri=CELEX:32017R0625.

[2] Winkler B., Maquet A., Reeves-Way E., Siegener E., Cassidy T., Valinhas de Oliveira T., Verluyten J., Jelic M., Muznik A. (2023). Fighting Fraudulent and Deceptive Practices in the Agri-Food Chain.

[3] Ulberth F. (2020). Tools to combat food fraud—A gap analysis. Food Chem..

[4] Knowledge Centre for Food Fraud and Quality of the European (KC-FFQ) (2023). Monthly Food Fraud Summary Reports. https://knowledge4policy.ec.europa.eu/food-fraud-quality/monthly-food-fraud-summary-reports_en.

[5] Official Journal of the European Union (OJEU) (2017). Commission Implementing Regulation (EU) 2017/1925 of 12 October 2017 amending Annex I to Council Regulation (EEC) No 2658/87 on the tariff and statistical nomenclature and on the Common Customs Tariff. OJ L 282/1, 31.10.2017. https://eur-lex.europa.eu/legal-content/EN/TXT/PDF/?uri=CELEX:32017R1925.

[6] Buthelezi N.M.D., Tesfay S.Z., Ncama K., Magwaza L.S. (2019). Destructive and non-destructive techniques used for quality evaluation of nuts: A review. Sci. Hortic..

[7] Eurostats (2023). Crop Production in EU Standard Humidity. https://ec.europa.eu/eurostat/databrowser/view/APRO_CPSH1__custom_8035705/default/table?lang=en.

[8] Luparelli A., Losito I., De Angelis E., Pilolli R., Lambertini F., Monaci L. (2022). Tree nuts and peanuts as a source of beneficial compounds and a threat for allergic consumers: Overview on methods for their detection in complex food products. Foods.

[9] Campmajó G., Saurina J., Núñez O. (2023). Liquid chromatography coupled to high-resolution mass spectrometry for nut classification and marker identification. Food Control.

[10] Ellis D.I., Brewster V.L., Dunn W.B., Allwood J.W., Golovanov A.P., Goodacre R. (2012). Fingerprinting food: Current technologies for the detection of food adulteration and contamination. Chem. Soc. Rev..

[11] Visciano P., Schirone M. (2021). Food frauds: Global incidents and misleading situations. Trends Food Sci. Technol..

[12] Aslam R., Sharma S.R., Kaur J., Panayampadan A.S., Dar O.I. (2023). A systematic account of food adulteration and recent trends in the non-destructive analysis of food fraud detection. J. Food Meas. Charact..

[13] Barreira J.C.M., Alves R.C., Casal S., Ferreira I.C.F.R., Oliveira M.B.P.P., Pereira J.A. (2009). Vitamin E profile as a reliable authenticity discrimination factor between chestnut (*Castanea sativa* Mill.) cultivars. J. Agric. Food Chem..

[14] Esteki M., Farajmand B., Amanifar S., Barkhordari R., Ahadiyan Z., Dashtaki E., Mohammadlou M., Heyden Y.V. (2017). Classification and authentication of Iranian walnuts according to their geographical origin based on gas chromatographic fatty acid fingerprint analysis using pattern recognition methods. Chemom. Intell. Lab. Syst..

[15] Rabadan A., Pardo J.E., Gómez R., Alvarruiz A., Álvarez-Ortí M. (2017). Usefulness of physical parameters for pistachio cultivar differentiation. Sci. Hortic..

[16] Brüning P., Haase I., Matissek R., Fischer M. (2011). Marzipan: Polymerase chain reaction-driven methods for authenticity control. J. Agric. Food Chem..

[17] Ballin N.Z., Mikkelsen K. (2016). Polymerase chain reaction and chemometrics detected several Pinus species including *Pinus armandii* involved in pine nut syndrome. Food Control.

[18] Caligiani A., Coisson J.D., Travaglia F., Acquotti D., Palla G., Palla L., Arlorio M. (2014). Application of 1H NMR for the characterisation and authentication of ‘‘Tonda Gentile Trilobata’’ hazelnuts from Piedmont (Italy). Food Chem..

[19] Eksi-Kocak H., Mentes-Yilmaz O., Boyaci I.H. (2016). Detection of green pea adulteration in pistachio nut granules by using Raman hyperspectral imaging. Eur. Food Res. Technol..

[20] Firmani P., Bucci R., Marini F., Biancolillo A. (2019). Authentication of ‘‘Avola almonds’’ by near infrared (NIR) spectroscopy and chemometrics. J. Food Compos. Anal..

[21] Taylan O., Cebi N., Yilmaz M.T., Sagdic O., Ozdemir D., Bulubaid M. (2021). Rapid detection of green-pea adulteration in pistachio nuts using Raman spectroscopy and chemometrics. J. Food Sci. Agric..

[22] Schmitt C., Bastek T., Stelzer A., Schneider T., Fischer M. (2020). Detection of peanut adulteration in food samples by nuclear magnetic resonance spectroscopy. J. Agric. Food Chem..

[23] Von Wuthenau K., Segelke T., Müller M.-S., Behlok H., Fischer M. (2022). Food authentication of almonds (*Prunus dulcis* Mill.). Origin analysis with inductively coupled plasma mass spectrometry (ICP-MS) and chemometrics. Food Control.

[24] Von Wuthenau K., Müller M.-S., Cvancar L., Oest M., Fischer M. (2022). Food authentication of almonds (*Prunus dulcis* mill.). Fast origin analysis with laser ablation inductively coupled plasma mass spectrometry and chemometrics. J. Agric. Food Chem..

[25] Teixeira M., Sousa C. (2019). A review on the application of vibrational spectroscopy to the chemistry of nuts. Food Chem..

[26] Panda B.K., Mishra G., Ramirez W.A., Jung H., Singh C.B., Lee S.-H., Le I. (2022). Rancidity and moisture estimation in shelled almond kernels using NIR hyperspectral imaging and chemometric analysis. J. Food Eng..

[27] Pannico A., Schouten R.E., Basile B., Romano R., Woltering E.J., Cirillo C. (2015). Non-destructive detection of flawed hazelnut kernels and lipid oxidation assessment using NIR spectroscopy. J. Food Eng..

[28] Torres I., Sánchez M.T., Vega-Castellote M., Pérez-Marín D. (2021). Fraud detection in batches of sweet almonds by portable near-infrared spectral devices. Foods.

[29] Mohammadi-Moghaddam T., Razavi S.M.A., Taghizadeh M. (2013). Applications of hyperspectral imaging in grains and nuts quality and safety assessment: A review. Food Meas..

[30] Gowen A.A., O’Donnell C.P., Cullen P.J., Downey G., Frias J.M. (2007). Hyperspectral imaging an emerging process analytical tool for food quality and safety control. Trends Food Sci. Technol..

[31] Lu Y., Saeys W., Kim M., Peng Y., Lu R. (2020). Hyperspectral imaging technology for quality and safety evaluation of horticultural products: A review and celebration of the past 20-year progress. Postharvest Biol. Technol..

[32] Pérez-Marín D., Garrido-Varo A. (2021). NIR Sensors for the In-Situ Assessment of Iberian Ham. Compr. Foodomics.

[33] Momtaz M.M., Bubli S.Y., Khan M.S. (2023). Mechanisms and health aspects of food adulteration: A comprehensive review. Foods.

[34] Dimitrakopoulou M.-E., Vantarakis A. (2023). Does Traceability Lead to Food Authentication? A Systematic Review from A European Perspective. Food Rev. Int..

[35] Faqeerzada M.A., Lohumi S., Joshi R., Kim M., Baek I., Cho B.-K. (2020). Non-targeted detection of adulterants in almond powder using spectroscopic techniques combined with chemometrics. Foods.

[36] Menevseoglu A., Entrenas J.A., Gunes N., Dogan M.A., Pérez-Marín D. (2024). Machine learning-assisted near-infrared spectroscopy for rapid discrimination of apricot kernels in ground almond. Food Control.

[37] Biancolillo A., De Luca S., Bassi S., Roudier L., Bucci R., Magrì A.D., Marini F. (2018). Authentication of an Italian PDO hazelnut (‘‘Nocciola Romana’’) by NIR spectroscopy. Environ. Sci. Pollut. Res..

[38] Sammarco G., Dall’Asta C., Suman M. (2023). Near infrared spectroscopy and multivariate statistical analysis as rapid tools for the geographical origin assessment of Italian hazelnuts. Vib. Spectrosc..

[39] Amendola L., Firmani P., Bucci R., Marini F., Biancolillo A. (2020). Authentication of Sorrento walnuts by NIR spectroscopy coupled with different chemometric classification strategies. Appl. Sci..

[40] Nardecchia A., Presutto R., Bucci R., Marini F., Biancolillo A. (2020). Authentication of the geographical origin of ‘‘Vallerano’’ chestnut by near infrared spectroscopy coupled with chemometrics. Food Anal. Methods.

[41] Cortés V., Barat J., Talens P., Blasco J., Lerma-García M.J. (2018). A comparison between NIR and ATR-FTIR spectroscopy for varietal differentiation of Spanish intact almonds. Food Control.

[42] Arndt M., Rurik M., Drees A., Ahlers C., Feldmann S., Kohlbacher O., Fischer M. (2021). Food authentication: Determination of the geographical origin of almonds (*Prunus dulcis* MILL.) via near-infrared spectroscopy. Microchem. J..

[43] Corona P., Frangipane M.T., Moscetti R., Lo Feudo G., Castellotti T., Massantini R. (2021). Chestnut cultivar identification through the data fusion of sensory quality and FT-NIR spectral data. Foods.

[44] Moscetti R., Berhe D.H., Agrimi M., Haff R.P., Liang P., Ferri S., Monarca D., Massantini R. (2021). Pine nut species recognition using NIR spectroscopy and image analysis. J. Food Eng..

[45] Genis H.E., Durna S., Boyaci I.H. (2021). Determination of green pea and spinach adulteration in pistachio nuts using NIR spectroscopy. LWT—Food Sci. Technol..

[46] Aykas D.P., Menevseoglu A. (2021). A rapid method to detect green pea and peanut adulteration in pistachio by using portable FT-MIR and FT-NIR spectroscopy combined with chemometrics. Food Control.

[47] Vega-Castellote M., Pérez-Marín D., Torres I., Moreno-Rojas J.M., Sánchez M.T. (2021). Exploring the potential of NIRS technology for the in situ prediction of amygdalin content and classification by bitterness of in-shell and shelled intact almonds. J. Food Eng..

[48] Torres-Rodríguez I., Sánchez M.T., Entrenas J.A., Vega-Castellote M., Garrido-Varo A., Pérez-Marín D. (2022). Hyperspectral Imaging for the detection of bitter almonds in sweet almond batches. Appl. Sci..

[49] Miaw C.S.W., Martins M.L.C., Sena M.M., De Souza S.V.C. (2022). Screening method for the detection of other allergenic nuts in cashew nuts using chemometrics and portable near-infrared spectrophotometer. Food Anal. Methods.

[50] Rovira G., Miaw C.S.W., Martins M.L.C., Sena M.M., De Souza S.V.C., Ruisánchez I., Callao M.P. (2022). In-depth chemometric strategy to detect up to four adulterants in cashew nuts by IR spectroscopic techniques. Microchem. J..

[51] Feng Z., Li W., Cui D. (2022). Detection of endogenous foreign bodies in Chinese hickory nuts by hyperspectral spectral imaging at the pixel level. Int. J. Agric. Biol. Eng..

[52] Arndt M., Rurik M., Drees A., Bigdowski K., Kohlbacher O., Fischer M. (2020). Comparison of different sample preparation techniques for NIR screening and their influence on the geographical origin determination of almonds (*Prunus dulcis* MILL.). Food Control.

[53] Vega-Castellote M., Sánchez M.T., Torres I., Pérez-Marín D. (2021). An innovative non-targeted control system based on NIR spectral information for detecting non-compliant batches of sweet almonds. Spectrochim. Acta Part A Mol. Biomol. Spectrosc..

[54] Lösel H., Shakiba N., Wenck S., Tan P.L., Arndt M., Seifert S., Hackl T., Fischer M. (2022). Impact of Freeze-Drying on the determination of the geographical origin of almonds (*Prunus dulcis* Mill.) by near-infrared (NIR) spectroscopy. Food Anal. Methods.

[55] Netto J.M., Honorato F.A., Celso P.G., Pimentel M.F. (2023). Authenticity of almond flour using handheld near infrared instruments and one class classifiers. J. Food Compos. Anal..

[56] Rovira G., Miaw C.S.W., Martins M.L.C., Sena M.M., De Souza S.V.C., Callao M.P., Ruisánchez I. (2023). One-class model with two decision thresholds for the rapid detection of cashew nuts adulteration by other nuts. Talanta.

[57] Ayvaz H., Temizkan R., Genis H.E., Mortas M., Genis D.O., Dogan M.A., Nazlim B.A. (2022). Rapid discrimination of Turkish commercial hazelnut (*Corylus avallana* L.) varieties using Near-Infrared Spectroscopy and chemometrics. Vib. Spectrosc..

[58] Shakiba N., Gerdes A., Holz N., Wenck S., Bachmann R., Schneider T., Seifert S., Fischer M., Hackl T. (2022). Determination of the geographical origin of hazelnuts (*Corylus avellana* L.) by near-infrared spectroscopy and a low-level fusion with nuclear magnetic resonance (NMR). Microchem. J..

[59] Huang B., Liu J., Jiao J., Lu J., Lv D., Mao J., Zhao Y., Zhang Y. (2022). Applications of machine learning in pine nuts classification. Sci. Rep..

[60] Gu X., Li Z., Li L., Ma N., Tu K., Song L., Pan L. (2018). Multisource fingerprinting for the region identification of walnuts in Xinjiang combined with chemometrics. J. Food Process Eng..

[61] Arndt M., Drees A., Ahlers C., Fischer M. (2020). Determination of the geographical origin of walnuts (*Juglans regia* L.) using near-infrared spectroscopy and chemometrics. Foods.

[62] Faqeerzada M.A., Perez M., Lohumi S., Lee H., Kim G., Wakholi C., Joshi R., Cho B.-K. (2020). Online application of a hyperspectral imaging system for the sorting of adulterated almonds. Appl. Sci..

[63] Porep J.U., Kammerer D.R., Reinhold C. (2015). On-line application of near infrared (NIR) spectroscopy in food production. Trends Food Sci. Technol..

[64] Liu Y., Pu H., Sun D.-W. (2017). Hyperspectral imaging technique for evaluating food quality and safety during various processes: A review of recent applications. Trends Food Sci. Technol..

[65] Müller-Maatsch J., van Ruth S.M. (2021). Handheld devices for food authentication and their applications: A review. Foods.

[66] Cortés V., Blasco J., Aleixos N., Cubero S., Talens P. (2019). Monitoring strategies for quality control of agricultural products using visible and near-infrared spectroscopy: A review. Trends Food Sci. Technol..

[67] Pérez-Marín D.C., Garrido-Varo A., Meyers R.A. (2023). Near-Infrared Spectroscopy and Chemometrics in Food and Agriculture. Encyclopedia of Analytical Chemistry.

[68] Shenk J.S., Workman J., Westerhaus M.O., Burns D.A., Ciurczac E.W. (2008). Application of NIR spectroscopy to agricultural products. Handbook of Near Infrared Analysis.

[69] Zhu C., Fu X., Zhang J., Qin K., Wu C. (2022). Review of portable near infrared spectrometers: Current status and new techniques. J. Near Infrared Spectrosc..

[70] Qin J., Chao K., Kim M.S., Lu R., Burks T.F. (2013). Hyperspectral and multispectral imaging for evaluating food safety and quality. J. Food Eng..

[71] Rinnan Å., van de Berg F., Engelsen S.B. (2009). Review of the most common pre-processing techniques for near-infrared spectra. Trends Anal. Chem..

[72] Boysworth M.K., Booksh K.S., Burns D.A., Ciurczak E. (2008). Aspects of multivariate calibration applied to near-infrared spectroscopy. Handbook of Near-Infrared Analysis.

[73] Riedl J., Esslinger S., Fauhl-Hassek C. (2015). Review of validation and reporting of non-targeted fingerprinting approaches for food authentication. Anal. Chim. Acta.

[74] Xu L., Yan S.-M., Cai C.-B., Yu X.-P. (2013). One-class partial least squares (OCPLS) classifier. Chemom. Intell. Lab. Syst..

[75] McGrath T.F., Haughey S.A., Patterson J., Fauhl-Hassek C., Donarski J., Alewijn M., van Ruth S., Elliot C.T. (2018). What are the scientific challenges in moving from targeted to non-targeted methods for food fraud testing and how can they be addressed? Spectroscopy case study. Trends Food Sci. Technol..

[76] Sádecká J., Jakubíková M., Májek P., Kleinová A. (2016). Classification of plum spirit drinks by synchronous fluorescence spectroscopy. Food Chem..

[77] Cortes C., Vapnik V. (1995). Support-vector networks. Mach. Learn..

[78] Oliveri P. (2017). Class-modelling in food analytical chemistry: Development, sampling, optimisation and validation issues—A tutorial. Anal. Chim. Acta.

[79] De Maesschalck R., Candolfi A., Massart D.L., Heuerding S. (1999). Decision criteria for soft independent modelling of class analogy applied to near infrared data. Chemom. Intell. Lab. Syst..

[80] Kemsley E.K. (1996). Discriminant analysis of high-dimensional data: A comparison of principal components analysis and partial least squares data reduction methods. Chemom. Intell. Lab. Syst..

